# Homeopathic treatment for prolonged postoperative coma: a case report

**Published:** 2017

**Authors:** G Vithoulkas, V Văcăraș, J Kavouras, AD Buzoianu, M Mărginean, D Văcăraș, S Cozma

**Affiliations:** * International Academy of Classical Homeopathy, Greece; ** Department of Neurosciences, “Iuliu Hațieganu” University of Medicine and Pharmacy, Cluj-Napoca, Romania; *** County Emergency Hospital Cluj, Cluj-Napoca, Romania; **** Department of Clinical Pharmacology, “Iuliu Hațieganu” University of Medicine and Pharmacy, Cluj-Napoca, Romania; ***** Department of Histology, “Iuliu Hațieganu” University of Medicine and Pharmacy, Cluj-Napoca, Romania; ****** Faculty of Medicine, “Iuliu Hațieganu” University of Medicine and Pharmacy, Cluj-Napoca, Romania

**Keywords:** postoperative coma, homeopathic treatment, Carbo Vegetabilis, Stanum metallicum, comatose patient

## Abstract

Coma is the state of unrousable unconsciousness. There are variations in the degree of coma and the findings and signs found on the patient’s clinical examination depend on the underlying cause of the disorder. The Glasgow Coma scale evaluates the best motor, verbal and eye answers of the patient. A patient is considered to be in a coma if his Glasgow Coma Scale is below 8 points.

The progress that we have made throughout the years has also led to complications that can culminate in a major catastrophe like death, permanent brain damage, coma. A study performed reached the conclusion that prior comorbidity, older age, intraoperative hypotension, and cardiovascular surgery may predispose patients to postoperative coma.

The article presents a case of postoperative coma treated successfully with homeopathy.

Although a rare complication, postoperative coma is a severe, death-leading condition, causing immense suffering on both the patient and the patient’s family. A multidisciplinary and thorough approach is necessary for these patients, but even after a well-conducted therapy, this condition leads to the death of the patient.

## Introduction

Coma is the state of unrousable unconsciousness [**1**]. There are variations in the degree of coma and the findings and signs found on the patient’s clinical examination depend on the underlying cause of the disorder [**2**]. Coma is produced by one of two important problems: one of them is morphologic, consisting of either a lesion of the brainstem and/ or diencephalon (primary of secondary to compression) or a widespread destructive lesion located in the hemispheres; the other one is submicroscopic or metabolic, having as a consequence the suppression of the neuronal activity in the cerebrum and reticular activating system [**2**]. The clinical examination of the comatose patient is somewhat limited by the unconsciousness of the patient, but the widely adopted Glasgow Coma Scale can be used in grading the patient’s state and his evolution in time [**3**]. The Glasgow Coma scale evaluates the best motor, verbal and eye answers of the patient [**4**]. A patient is considered to be in a coma if his Glasgow Coma Scale is below 8 points [**5**].

Seriously impaired states of consciousness, regardless of the cause, are often fatal because they add their own particular burdens to the primary disease causing it [**6**]. Therefore, the management of such a patient must be extremely prompt and well conducted, keeping in mind the fact that a comatose patient’s state can never be considered fully stable.

The management of a comatose patient should include the protection against hypoxia and hypoventilation by administrating oxygen or even endotracheal intubation and assisted ventilation, keeping fluid and electrolytes balanced, maintaining a proper glycemic level, avoiding gastric haemorrhage and excessive gastric secretion, avoiding aspiration pneumonia by using a gastric tube and endotracheal intubation, avoiding deep vein thrombosis by using low molecular weight heparin and intermittent pneumatic compression boots. The management must be individualized and very well adapted to each patient’s needs [**1**,**2**,**7**].

Nowadays, the progress that medicine has sustained has brought new challenges as well as benefits. The advanced technology has allowed the development of new surgical procedures and techniques, as well as new ways of inducing and maintaining the anaesthesia in order to be able to perform them. This progress that we have made throughout the years has also led to complications that can culminate in a major catastrophe like death, permanent brain damage, coma [**6**,**8**,**9**]. The severity of the postoperative coma is bidirectional: on one hand, the patient’s suffering and on the other hand the family’s suffering that is subjected to the harrowing experience of seeing the patient in a comatose state [**8**].

Because of the high impact postoperative coma has on the both medical world and the society in general, researchers have started studying this subject more thoroughly.

A case-controlled study has been performed by a group of researchers at Mayo Clinic having the purpose of identifying predictive factors for postoperative coma or stupor and examining the value of neuroimaging techniques in elucidating structural brain damage. They have reached the conclusion that prior comorbidity, older age, intraoperative hypotension, and cardiovascular surgery may predispose patients to postoperative coma. Widespread structural ischemic brain damage was often documented by neuroimaging. Metabolic causes for coma were uncommon [**10**].

The largest study on postoperative coma has analyzed data from 858 606 patients. Its purpose was to determine the incidence, risk factors and impact of postoperative coma in a large patient population. The incidence of postoperative coma was 0.06%. Multivariate analysis revealed the following independent predictors: liver disease, systemic sepsis, age above 63 years old, renal disease, emergency operation, cardiac disease, hypertension, prior neurological disease, diabetes mellitus. These predictors were incorporated into a risk index classification; odds ratios for postoperative coma increased from 2.5 with one risk factor to 18.4 with three. Coma was associated with 74.2% all-cause mortality [**11**].

As postoperative coma is a fairly rare event, but also a fatal one in most cases, families and doctors have started considering alternative therapies as a final resort to regain their loved ones and their patients back.

The article presents a case of postoperative coma treated successfully with homeopathy.

## Case report

An 81-year-old female patient was admitted in July 2015 to the Cardiovascular Surgery Department of a hospital in Bucharest for an aortic valve replacement surgery. 

The patient had a history of mild hypertension, insulin-dependent type 2 diabetes, coronary artery disease, congestive heart failure NYHA 2, severe aortic stenosis, moderate mitral regurgitation, mild pulmonary hypertension, bilateral carotid atheromatosis with a 50% stenosis of the left internal carotid artery, complete right mastectomy for breast cancer (at that moment in remission).

After a preoperative evaluation and preparation, the surgery was completed with the replacement of the aortic valve with a bioprosthesis (Medtronic Hancock II Ultra no. 23) and myocardial revascularization by using a double aortic-coronary bypass.

The post-operatory evolution was a good one in terms of the heart disease. However, the patient did not regain consciousness after the anaesthesia, maintaining a deep comatose state (GCS 7 points – E1V2M4).

A brain CT was performed the third day postoperatively, showing no recent ischemic or haemorrhagic cerebral lesions, moderate diffuse cerebral atrophy and carotid atheromatosis. 

After the surgery, the patient was admitted to the Intensive Care Unit and was treated by using a multidisciplinary approach. The patient was treated with inotropic, antiarrhythmic, and diuretic drugs, insulin and antidiabetic drugs were used in order to keep the blood sugar levels under control. The patient was kept hydrated and the electrolytes balanced by using an i.v. line, prophylaxis for deep vein thrombosis, and pulmonary thromboembolism was performed by using low molecular weight heparin. Prophylaxis for bedsores was also performed by using a pressure relieve air mattress.

The patient went into acute respiratory distress, needing mechanical ventilation in order to maintain oxygenation. 

Despite these complex and correctly performed therapeutic efforts, the patient did not regain consciousness and was still in a deep coma in the fourteenth day post-operatory (GCS 7 points – E1V2M4), without having a confirmed medical explanation. 

At that point, the patient’s family requested a consult from a homeopathic specialist.

The homeopathic examination, which was performed in the fourteenth day postoperatively, revealed the following: old, comatose, tranquil patient, with pale and cold skin, with the need to uncover herself (the few movements that she made with her hands were to remove her blanket and clothes, as if she wanted more air – “thirst for air”), abdominal distension, and bloating.

The thorough evaluation of the patient and the analysis of her symptoms led us to the remedy most appropriate for this critical situation – Carbo Vegetabilis. 

Homeopathic treatment was initiated the same day, by using Carbo Vegetabilis 200CH 7 granules twice a day, administered diluted in 20ml of water by using a nasogastric tube. 

The patient’s evolution was spectacular. The next day after the initiation of the treatment (fifteenth day postoperatively) the patient was in a superficial coma (GCS 11 points – E2V4M5), and the following day she regained consciousness. Carbo Vegetabilis was administered in the same dose for a total of five days (including the nineteenth day postoperatively).

After these five days, the case was reassessed from a homeopathically point of view and the second evaluation revealed the following: severely dyspnoeic patient (even talking caused exhaustion) with pale skin, severe fatigue aggravated by the slightest movements, a weakness sensation located in the chest area, extreme lack of energy, the wish “to be left alone”.

Considering the state of general exhaustion the patient was in at that moment and her lack of energy, the homeopathic treatment was changed to a new remedy: Stanum metallicum 30CH 7 granules administered sublingually twice a day for a week.

After the administration of the second remedy, the patient’s general condition improved dramatically: she started eating, she was able to get up in a sitting position with only little help, her fatigue diminished significantly.

The patient was then transferred to a recovery clinic in Cluj-Napoca in order to continue the cardiovascular recovery treatment. During her three-week admission in the clinic, she followed an individualized cardiovascular recovery program, which led to her ability to walk short distances with minimal support and has was released from the hospital in September 2015.

The following weeks after release, the patient recovered almost entirely, both physically and mentally. She was able to retake her place in her family and in society in general.

## Discussion

The homeopathic approach is completely different from the classical, allopathic one. If the allopathic medicine has the tendency to suppress any symptom, homeopathy uses these symptoms in order to choose the correct remedy (the remedy that would produce the same symptoms in a healthy person). Given to a sick person, this remedy will activate that person’s own defense mechanisms, which will grow stronger and lead that person’s organism to self-healing [**12**,**13**].

The healing depends mostly on the organism’s level of health when the illness occurs. If the organism is in one of the A and B groups (levels 1-6), one can get to healing by the administration of either one or a succession of 2, 3, 4 correct homeopathic remedies. If the organism is in one of the C and D groups (levels 7-12), the chances of healing are limited. In these cases, we can only ease the patient’s suffering, or, if the patient is in the C group, climb up the level of health and even reach healing by the correct administration of several remedies, but during a longer period of time [**14**].

The symptoms taken into account in this case were the unconsciousness, the pale skin, the cyanotic lips and finger tips, the “thirst for air”- the wish to uncover herself even if the body temperature was low, the abdominal distension. The remedy of choice was Carbo vegetabilis, a remedy known for its usefulness in critical situations, having as a starting point especially circulatory-cardiac problems [**15**].

The patient’s condition improved in a spectacular manner: she regained consciousness. However, she was in a state of extreme physical exhaustion caused by the simple acts of talking, eating, or slightly moving her limbs. That state led to the administration of the following remedy: Stanum metallicum. It is a remedy known for its usefulness in extreme physical exhaustion states, where even the slightest efforts lead to the patient feeling worse [**16**,**17**].

However, in this case, considering the patient’s medical history, her age, and her multiple illnesses, homeopathy was used as a complementary treatment to the classical, allopathic treatment the patient underwent, its primary purpose being to aid the body in activating its own healing mechanisms [**12**].

Considering the severity of a postoperative complication such as coma and the impact it has not only on the patient, but also on the patient’s family, we believe that homeopathy should be taken into consideration as a viable solution for this type of situations.

## Conclusion

Although a rare complication, postoperative coma is a severe, death-leading condition, causing immense suffering on both the patient and the patient’s family. A multidisciplinary and thorough approach is necessary for these patients, but even after a well-conducted therapy, this condition leads to the death of the patient. 

We presented a case of an elderly patient with many severe illnesses, who recovered from a prolonged postoperative coma with the aid of homeopathy. 

We believe that this case should bring a new light on the use of complementary therapy such as homeopathy in the management of the patients suffering from severe conditions.

**Acknowledgement**

George Vithoulkas, on one hand, and Vitalie Văcăraș, on the other hand, have equally contributed to the present paper.

## References

[1] Manji H, Connolly  S, Dorward N, Kitchen  N, Mehta A, Wills  A (2007). Oxford Handbook of Neurology.

[2] Ropper AH, Samuels  MA, Klein JP (2014). Adams and Victor’s Principles of Neurology.

[3] Szatmari S, Szasz  JA (2007). Urgente neurologice.

[4] Longmore M, Wilkinson  IB, Davidson  EH, Foulkes  A, Mafi  AR (2010). Oxford Handbook of Clinical Medicine.

[5] Leacche M, Winkelmayer  WC, Subroto  P (2006). Predicting survival in patients requiring renal replacement therapy after cardiac surgery. Annal of Thoracic Surgery.

[6] De Eelco FMW (2014). The Comatose Patient.

[7] Fauci K, Longo H, Loscalzo J (2014). Harrison Principles of Internal Medicine.

[8] Sandhu K, Dash  HH (2004). Anaesthesia related neurological complications. NDHU, DASH Indian Journal of Anaesthesia.

[9] Posner JB, Saper CB, Schiff ND, Plum F (2007). Plum and Posner’s Diagnosis of Stupor and Coma.

[10] Gootjes EC, Wijdicks  EF, McClelland  RL (2005). Postoperative stupor and coma. Mayo Clinic Proceedings.

[11] Newman J, Blake  K, Fennema  J, Harris  D, Shanks A, Avidan  MS, Kelz  MB, Mashour  GA (2013 ). Incidence, predictors and outcomes of postoperative coma: An observational study of 858 606 patients. European Journal of Anaesthesiology.

[12] (2015 ). The Society of Homeopaths [Internet].

[13] Vithoulkas G, Woensel E (2010). Levels of Health.

[14] Vithoulkas G, Woensel E (2010). Levels of Health.

[15] Vithoulkas G (1990). Essence of Materia Medica Viva.

[16] Vithoulkas G (1990). Essence of Materia Medica Viva.

[17] (2015 ). Webhomepath.

